# Metacommunity theory for transmission of heritable symbionts within insect communities

**DOI:** 10.1002/ece3.5754

**Published:** 2019-12-02

**Authors:** Joel J. Brown, Joseph R. Mihaljevic, Lauren Des Marteaux, Jan Hrček

**Affiliations:** ^1^ Faculty of Science University of South Bohemia Ceske Budejovice Czech Republic; ^2^ Biology Centre of the Czech Academy of Sciences Institute of Entomology Ceske Budejovice Czech Republic; ^3^ School of Informatics, Computing, and Cyber Systems Northern Arizona University Flagstaff AZ USA

**Keywords:** bacteria, dispersal, heritable, insect, metacommunity, microbiome, species interactions, symbiont, transmission

## Abstract

Microbial organisms are ubiquitous in nature and often form communities closely associated with their host, referred to as the microbiome. The microbiome has strong influence on species interactions, but microbiome studies rarely take interactions between hosts into account, and network interaction studies rarely consider microbiomes. Here, we propose to use metacommunity theory as a framework to unify research on microbiomes and host communities by considering host insects and their microbes as discretely defined “communities of communities” linked by dispersal (transmission) through biotic interactions. We provide an overview of the effects of heritable symbiotic bacteria on their insect hosts and how those effects subsequently influence host interactions, thereby altering the host community. We suggest multiple scenarios for integrating the microbiome into metacommunity ecology and demonstrate ways in which to employ and parameterize models of symbiont transmission to quantitatively assess metacommunity processes in host‐associated microbial systems. Successfully incorporating microbiota into community‐level studies is a crucial step for understanding the importance of the microbiome to host species and their interactions.

## Graphical abstract

We present the metacommunity framework as a method to include the transmission of symbiotic bacteria within insect communities. We provide an overview of both symbionts and the metacommunity framework, before suggesting multiple scenarios of how to employ experimental studies on this topic and demonstrate how to parameterize models for symbiont transmission.

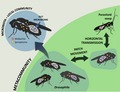

## 1 INTRODUCTION

Microbial organisms readily live in symbiosis with their host, often forming communities referred to as a microbiome. The microbiome is a broad term that defines the microscopic, symbiotic organisms associated with a particular host, and which can provide essential services for their host (e.g., aiding in immunity and digestion), thus providing insight into the health of the host organism (Fierer et al., 2012). The microbiome can have strong influence on the ecological niche occupied by the host species (Henry, Maiden, Ferrari, & Godfray, 2015; Hoffmann, Ross, & Rašić, 2015), and these symbiont‐induced changes to host ecology have increasingly clear impacts on the identity, strength, and outcome of interactions between hosts within communities (Berry & Widder, 2014; Cusumano et al., 2018; Frago, Dicke, & Godfray, 2012; Frago et al., 2017; Hrček, McLean, & Godfray, 2016; McLean, Parker, Hrček, Henry, & Godfray, 2016; Oliver, Smith, & Russell, 2014; Xie, Vilchez, & Mateos, 2010; Zhu et al., 2018). Understanding the spatiotemporal distribution and function of symbiont communities therefore has implications for basic and applied ecological theory.

A promising framework under which symbiont community dynamics can be explored is the metacommunity. An ecological community is an assemblage of multiple species living in a specified place and time with the potential to engage in ecological interactions (Agrawal et al., 2007; Vellend, 2010). A metacommunity scales up from this definition, linking multiple communities together via dispersal of multiple potentially interacting species (reviewed in Leibold et al., 2004). The crucial element of metacommunity theory, and where it differs from standard community ecology, is the exploration of how local and regional processes interact to influence patterns of community composition across space and time (Leibold & Chase, 2017). The metacommunity framework has been most frequently applied to natural communities defined by discrete habitat patches (such as alpine meadows and aquatic pools; Leibold & Chase, 2017; Logue, Mouquet, Peter, & Hillebrand, 2011; Mihaljevic, 2012). The relevance of studying organisms in a community context applies at both microbe and host levels, with the metacommunity concept allowing us to consider both levels simultaneously. Logue et al. (2011) found that empirical metacommunity studies lacked data on trophic interactions, in addition to lacking experimental work from terrestrial systems. We believe that symbiont–host metacommunities are ripe to fill these research gaps and provide further insight into currently unanswered questions in symbiosis research and community ecology.

Specifically, we believe that the metacommunity concept will help us explore (a) symbiont vertical and horizontal transmission (dispersal), and (b) the influence of symbiont–symbiont interactions on their transmission and phenotype. The study of symbiont dispersal must take into account how local processes, such as interactions between multiple symbionts, shape symbiont populations sizes and density‐dependent dispersal (transmission). From the host community perspective, we must account for the effects of symbionts present in the local community and the dispersal processes that facilitate symbiont migration into a host. The importance of symbiotic bacteria to a wide variety of insect hosts (Box 1) suggests that symbiont communities and the processes that structure them are crucial for understanding the biology of the host insects, both as single entities and in the context of the wider insect community (Ferrari & Vavre, 2011; Hrček et al., 2016; McLean et al., 2016). The metacommunity concept provides us with a necessarily broad approach that includes local and regional processes. In this review, our use of the term “symbiont” refers broadly to commensal, mutualistic, or parasitic bacteria that exist in close physical association with their host. We focus on insect–bacteria associations because insects are often a model system for both community ecology and symbiosis studies, and bacteria are common members of microbiomes that have a well‐documented history of affecting insect host ecology (Bourtzis et al., 2014; Corbin, Heyworth, Ferrari, & Hurst, 2017; Crotti et al., 2012; Ross et al., 2017) and are relatively easy to identify with modern molecular methods. More specifically, we focus on the heritable bacteria that contextually transition between being beneficial and detrimental for their host. This includes both facultative endosymbionts (those found within host cells and hemolymph) and the symbiotic bacteria associated with the gut (commonly referred to as the “gut microbiome”).

Recently, several studies have advocated for the application of metacommunity theory to understand the dynamics of symbiotic and/or pathogenic organism communities within and among their hosts (Borer, Laine, & Seabloom, 2016; Costello, Stagaman, Dethlefsen, Bohannan, & Relman, 2012; Fierer et al., 2012; Johnson, Roode, & Fenton, 2015; Mihaljevic, 2012; Miller, Svanbäck, & Bohannan, 2018; Seabloom et al., 2015). However, most have proposed conceptual models without sufficient advice on how to empirically or quantitatively assess such dynamics. Furthermore, most of the empirical approaches that have been suggested are in the realm of inferring processes from static patterns of community composition. More powerful approaches involve an integration of longitudinal data and dynamical models to infer the dominant, mechanistic processes that influence community composition over space and time. Here, we extend the metacommunity concept to heritable symbionts, specifically considering their transfer (i.e., dispersal). The concepts discussed here will apply to other symbioses (e.g., plants and endophytic fungi, vertebrates and their organ microbiomes, or insect–virus–plant systems), but for the sake of clarity we focus on insect–bacteria associations. We believe that using a metacommunity approach will facilitate a deeper understanding of insect–symbiont systems, by focusing on the local and regional ecological processes that influence symbiont community assembly, the process of symbiont dispersal via horizontal and vertical transmission, and the consequences for the host organisms.

### 1.1 Objectives

**Figure 1:**
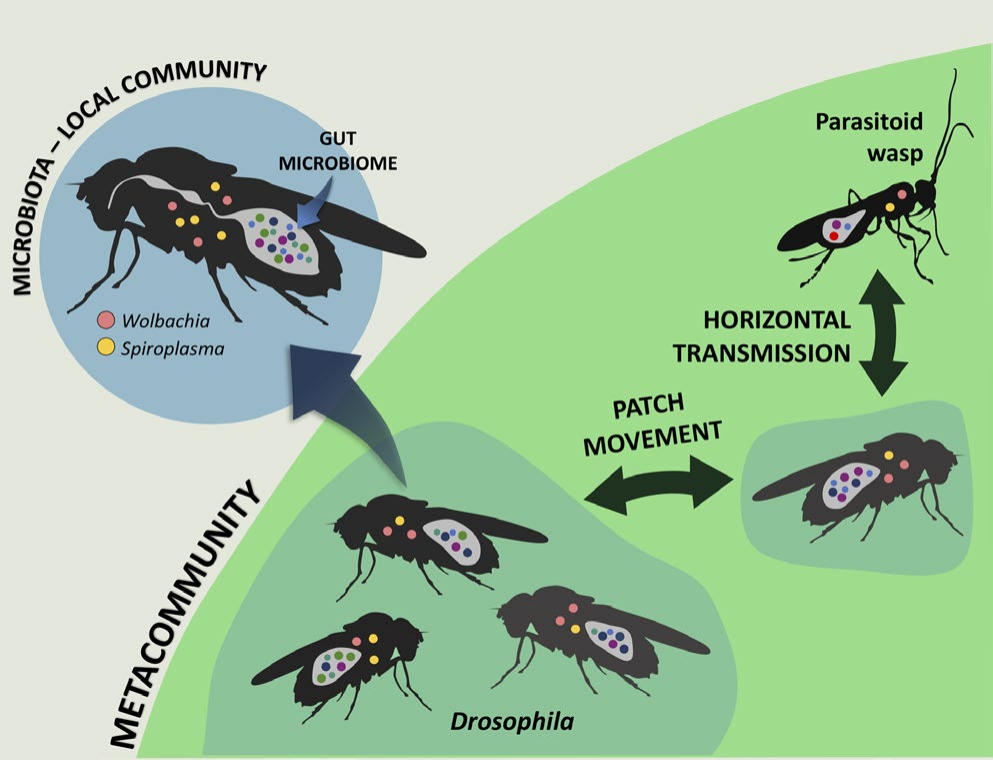
Applying the metacommunity concept to microbial communities of insects, in this case a community of hosts (*Drosophila*) and parasitoids. Each individual insect is a “patch” that harbors a local community of endosymbiotic bacteria. The green area represents the regional metacommunity of hosts. Bacteria can be present both within the gut and inside host cells and hemolymph (with *Wolbachia* and *Spiroplasma* as specific examples of the latter category). Differently colored circles within an insect each represent a different bacterial genus. Arrows indicate horizontal transmission (dispersal) of bacteria among local communities (host microbiomes). This diagram represents one of multiple ways to apply metacommunity theory to host–symbiont systems; see Table 1 scenarios B‐E for alternative approaches

In this review, we explore how symbiosis research can be fruitfully integrated with metacommunity theory to advance both fields. First, we provide an overview of the influence of microbial communities on the biology and interactions of their insect hosts (Box 1, see also McLean et al., 2016 and Corbin et al., 2017 for recent reviews on symbiotic bacteria in insect communities). This is followed by an examination of microbial transmission and its importance for host communities. We then propose how and why the metacommunity concept should be considered for advancing our understanding of symbiont transmission within insect–microbe networks, and highlight the future directions these studies could take (Figure 1, Boxes 2 and 3, Table 1). Specifically, we introduce a mathematical modeling framework and give concrete examples of how to conduct experiments with insect study systems to parameterize these models and better understand the roles of metacommunity processes in structuring symbiont communities. Our aim is to stimulate ideas for combining research on the microbiome and host community ecology. We present the metacommunity framework as a possible method to achieve this, but recognize that other macroecological approaches could be complementary. As we will outline in this paper, the importance of the microbiome to host biology suggests that microbiomes should be considered when studying communities of host organisms.

## 2 INSECT‐ASSOCIATED SYMBIOTIC BACTERIA

For the purpose of this paper, we focus on both endosymbiotic and symbiotic gut bacteria within insect hosts. Endosymbionts (bacteria living within the host's cells or hemolymph) can be obligate (primary) symbionts and thus necessary for host survival, or facultative (secondary) symbionts which are often helpful but not required for host survival. Obligate symbiont transmission is predictable because it is inextricably linked to host reproduction, whereas transmission of facultative symbionts is much more variable, leading to fluctuation in their abundance and diversity (explained further in “Microbiome transmission,” below). Pea aphids (*Acyrthosiphon pisum*) have the best known endosymbiont community to date, with a total of seven (up to four can be present in one individual). *Drosophila* species have a maximum of two known endosymbionts while spiders, another well‐studied invertebrate group, have a total of five (Goodacre, 2011). Gut symbionts are often collectively referred to as the gut microbiome. Insects have highly variable gut symbiont species richness (Christian, Whitaker, & Clay, 2015) which is largely dependent on the diet and lifestyle of the host species (Blum, Fischer, Miles, & Handelsman, 2013; Kaltenpoth, Winter, & Kleinhammer, 2009; Martinson, Douglas, & Jaenike, 2017; Nováková et al., 2017). For example, saproxylic beetles and termites have demonstrably large and diverse gut microbiomes based on their consumption of decaying wood (i.e., cellulose; Ohkuma, 2008), whereas some caterpillars have relatively depauperate gut microbiomes because they only feed on a single host‐plant species (Hammer, Janzen, Hallwachs, Jaffe, & Fierer, 2017).

**Figure 2:**
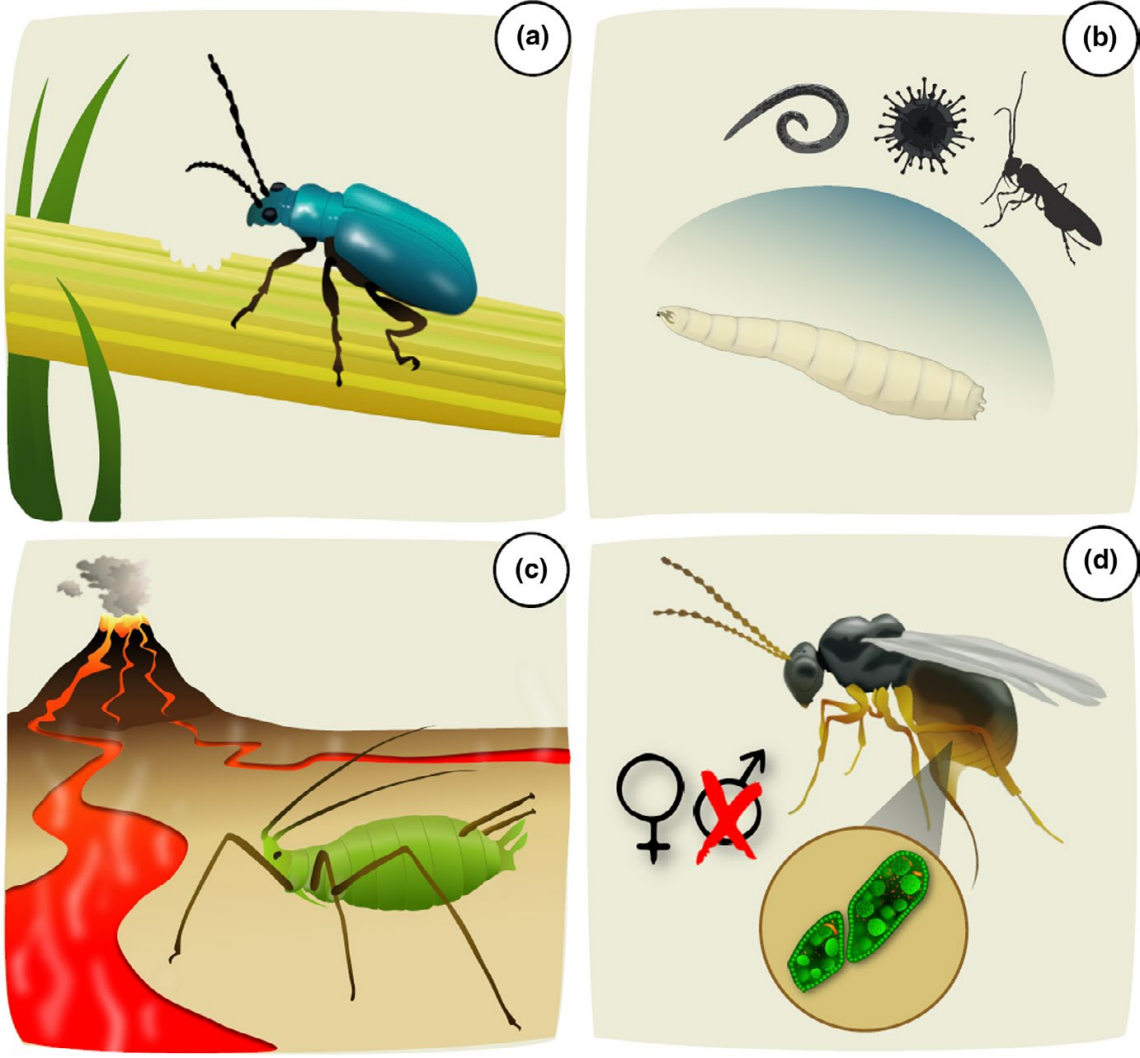
Representative examples of how microbial symbionts influence insect host ecology, physiology, and health. (a) novel symbioses can facilitate host insect feeding on a new food source; (b) the presence of specific microbes can protect a host against natural enemies such as parasitoids, fungi, and nematodes; (c) symbionts can modify host thermal tolerance in both positive and negative ways; and (d) some symbionts, like *Wolbachia* and *Spiroplasma,* manipulate host sex ratios by male‐killing, genetic feminization, and by inducing cytoplasmic incompatibility

Symbiont dispersal (their transmission between hosts, see “Microbial transmission” below) is an important determinant of microbiome diversity within the host (Henry et al., 2013). The profile of symbiotic bacteria within a particular host can in turn influence various aspects of host biology, including feeding behavior, sex ratios, resistance to parasitism, and thermal tolerance (Figure 2; Box 1; see also Feldhaar, 2011; Ferrari & Vavre, 2011; Ottman et al., 2012; McLean et al., 2016; Martino, Ma, & Leulier, 2017). This interaction between the host and symbiont community therefore ultimately shapes the spatial distributions of insects and their inter‐ or intraspecific interactions, with cascading effects on community and broader ecosystem processes (Chandler, Lang, Bhatnagar, Eisen, & Kopp, 2011; Frago et al., 2012, 2017; Hrček et al., 2016).

### 2.1 Interactions within microbial communities

Interactions between the microbial species in an individual host impact both the host and the function of the microbiome itself. Foster and Bell (2012) reported that the majority of interactions between microbial species were competitive, and thus classified as negative. Competition between gut microbiome species is also associated with a reduction in cooperation, which results in a decrease in community productivity (i.e., an inability to digest as efficiently; Oliveira, Niehus, & Foster, 2014). Ecological modeling by Coyte, Schluter, and Foster (2015) showed that competition between microbes facilitated stability within microbial communities, to the extent that the stabilizing effects were sufficient to counteract any destabilizing effects caused by increased cooperation or diversity. Based on this evidence, species interactions (such as competition) within a microbial community have both positive and negative effects and are therefore crucial factors to consider when analyzing animal–microbe symbioses. When viewed from a metacommunity perspective, there is strong potential for interactions between symbionts to affect their distribution among insect hosts, and consequently the biology and interactions of their hosts as well.

Microbes can also facilitate the establishment of other microbial species within the microbiome community. Some symbiont species are more likely to occur in coinfections; for example, *Fukatsui symbiotica* (Manzano‐Marín, Szabó, Simon, Horn, & Latorre, 2017) is a facultative symbiont that is almost always found in coinfection with *Hamiltonella defensa* in aphids feeding on *Medicago sativa* in Europe and North America. McLean et al. (2018) found stable coinfections to be possible between multiple combinations of different aphid symbionts and even between multiple strains of the same symbiont, *H. defensa*. Similarly, in a long‐term study of aphid symbiont communities, Rock et al. (2017) found that the bacteria *Serratia symbiotica* and *Rickettsiella viridis* co‐occurred more often than expected, a phenomenon that was explained by their ability to promote each other's transmission to the next host generation. *Wolbachia* is also positively associated with *Spiroplasma* within *Drosophila neotestacea* (Fromont, Adair, & Douglas, 2019).

## 3 MICROBIAL TRANSMISSION

In the context of metacommunity theory, the dispersal of organisms among habitat patches can influence local interactions and ultimately affect the community composition across space. For symbionts, dispersal can occur across host generations, between individuals of a single species, and across multiple species and trophic levels. Symbiont dispersal depends on two main factors: the ability to transmit from one host to the next and the ability to successfully establish within the new host. Symbionts can be transmitted vertically (parent to offspring) but also horizontally (between individuals or via the environment; Caspi‐Fluger et al., 2012; Haselkorn, Markow, & Moran, 2009; Hosokawa et al., 2016; Jaenike, 2009; Li et al., 2017).

### 3.1 Vertical transmission

Vertical transmission is typically the dominant form of symbiont dispersal (especially among endosymbionts) and occurs primarily from mother to offspring, although rare cases of paternal transmission have been documented (Moran & Dunbar, 2006). Gut microbes are generally not considered to be heritable, but are often transmitted from parent to offspring either directly or through the environment (Estes et al., 2013; Shukla, Vogel, Heckel, Vilcinskas, & Kaltenpoth, 2018). Some insects, especially true bugs, even display specialized behaviors that transmit their bacteria to offspring (e.g., via parental postoviposition secretions; Kaltenpoth et al., 2009). This "indirect inheritance" of gut microbes can be crucial to the well‐being and functioning of the new generation, and therefore influences how individuals of the new generation interact in their communities.

### 3.2 Horizontal transmission

Horizontal transmission of a symbiont includes transmission via host‐to‐host contact (either inter‐ or intraspecific) as well as acquisition from the environment. The precise mechanisms are poorly known, but it is widely presumed that horizontal transmission is a key mode of symbiont dispersal (Henry et al., 2013). Evidence for this presumption is provided by broad analyses of endosymbiont distribution. For example, strains of *Wolbachia* (the most common endosymbiotic bacteria in insects) are not distributed throughout insect clades in a phylogenetically or geographically clustered way, suggesting multiple horizontal transfer events in which the endosymbiont jumped from one species to another of distant relation (Smith et al., 2012). In the case of *Wolbachia,* multiple acquisitions from the environment are unlikely because the symbiont cannot survive outside hosts. A similar lack of phylogenetic clustering has been shown for incidences of symbiont infection within aphids (Henry et al., 2015). On an intraspecific level, dispersal of symbionts can be viewed as a pool of adaptations available for selection when they are advantageous to their host (Henry et al., 2013). The mechanism of horizontal transmission supported by the most evidence is that of "the dirty needle effect," whereby an uninfected parasitoid picks up a bacterium when parasitizing an infected host and then transmits the bacterium to a new uninfected host in a second parasitism event (Ahmed et al., 2015; Gehrer & Vorburger, 2012). Gehrer and Vorburger (2012) demonstrated this phenomenon by allowing parasitoids to attack an aphid clonal line possessing *H. defensa* and then attacked aphids of a “recipient” clonal line, allowing any survivors of attempted parasitism to mature and reproduce. In a number of cases, the offspring of these “recipient” aphids tested positive for *H. defensa*. Ahmed et al. (2015) showed that the parasitoids of *Bermisia tabaci* whiteflies picked up *Wolbachia* from infected hosts on their mouthparts and ovipositors, and could then effectively transmit *Wolbachia* to new hosts for 2 days.

### 3.3 Establishment

Successful establishment of a symbiont within a novel host is an important component of symbiont transmission. A symbiotic bacterium could survive for a short period of time in a novel host but may ultimately fail to reproduce or survive in the long term. Therefore, an important biological distinction must be made between the occurrence of a horizontal transmission event and successful symbiont establishment. Establishment success is an important filter for interspecific transmission, and as a result, the establishment rate of symbionts is highly variable. Gehrer and Vorburger (2012) reported an estimated 8.6% rate of establishment for *H. defensa* that was transmitted via parasitoids (the dirty needle effect), whereas Ahmed et al. (2015) found a 93.8% transmission rate of *Wolbachia* via parasitoids during their experiment. In another example, Łukasik et al. (2015) found that *H. defensa* established more easily when it was transferred from an individual of the same species as the recipient host. Similarly, establishment was most successful when the introduced symbiont strain was more closely related to the pre‐existing symbiont strain in the host (also shown by Tinsley & Majerus, 2007). In some cases (and perhaps more often than not), introduction of a symbiont into a novel host species can severely reduce host viability (Hutchence, Fischer, Paterson, & Hurst, 2011; Nakayama et al., 2015). The mechanisms underlying these harmful introductions have yet to be fully explored, but the consensus hypothesis is that novel symbiont failure is not simply a product of host responses to infection. Obadia et al. (2017) determined that stochastic factors were the main drivers of gut microbiome establishment, based on alternative stable states of colonization and high between‐individual variability in composition. Therefore, gut microbiome establishment is an inherently difficult process to predict.

### 3.4 Transmission of function

In cases where a symbiont successfully transfers and establishes in a novel host, it is still not guaranteed that it will provide the same function(s) in the new host. A symbiont that confers a protective phenotype for one host genotype may (Parker, Hrček, McLean, & Godfray, 2017) or may not (Chrostek et al., 2013) provide the same benefit in other host genotypes or species (Veneti et al., 2012). Transmission of symbiont function (or phenotype) is an important reason to integrate the microbiome with host community ecology. Particularly in cases where symbionts facilitate host defense (see Box 1), transmission of symbiont function can have drastic effects on host survival and interactions with other species (e.g., *Regiella insecticola* protects aphids against parasitoids; Vorburger, Gehrer, & Rodriguez, 2010). In the case of the dirty needle effect described in the “horizontal transmission” paragraph above, *B. tabaci* whiteflies that received *Wolbachia* from a wasp had subsequently increased survival and reduced development times, a tangible benefit for the host that received the symbiont (Ahmed et al., 2015). Parker et al. (2017) demonstrated that the strength of protective phenotypes conferred by transfer of *Regiella* varied with host genotype, providing further evidence for the complexities of context dependency in host–symbiont interactions. Similarly, Veneti et al. (2012) showed that a male‐killing *Wolbachia* strain did not transfer that phenotype when introduced to novel hosts, despite the novel hosts being sister species of the original host. Variation in phenotype transfer is likely a product of host and symbiont genotypes, and how they have evolved together. The function of each symbiont is therefore important to consider when discussing the possibilities of phenotype transfer to novel hosts. For example, symbiont‐induced male‐killing can transfer more readily (Ahmed et al., 2015) than defense against parasitoids (Gehrer & Vorburger, 2012).

Transmission of function is a more intricate and difficult process to consider when the particular function in question is a direct result of community complexity. For example, immunity or digestion can be improved with a more complex microbiome (Chaplinska, Gerritsma, Dini‐Andreote, Salles, & Wertheim, 2016). Loss of microbiome complexity and species abundance, often referred to as dysbiosis, is shown to have negative health effects in insects, corals, and humans (Bajaj et al., 2014; Hamdi et al., 2011; Petersen & Round, 2014; Raymann, Shaffer, & Moran, 2017; Sansone et al., 2017), among others. Currently, it is unclear whether keystone species (i.e., those required for healthy gut function in the host) occur within microbiome communities. Experimental species removal (or insertion) from the microbiome could be one approach to determine whether particular species play disproportionately important roles for host function.

Many facultative symbionts exist at intermediate abundance within host populations as a result of balancing selection and seasonal fluctuation (Oliver et al., 2014). In certain scenarios, hosts experience ecological and evolutionary costs from carrying symbionts. These costs can be subtle, yet significant, for host survival (Polin, Simon, & Outreman, 2014; Vorburger, Ganesanandamoorthy, & Kwiatkowski, 2013). Fitness costs also have important implications for the transmission of symbionts. The line separating a beneficial symbiont from one that is detrimental to its host is often blurred and context‐dependent. For example, a facultative symbiont that protects against a parasitoid can also reduce the host's competitive ability in the absence of said parasitoid or in different abiotic environments (Oliver, Campos, Moran, & Hunter, 2008), subsequently reducing host longevity (Vorburger & Gouskov, 2011) and fecundity (Simon et al., 2011). This variable selection pressure means that facultative symbionts will not always be transmitted, vertically or horizontally.

The effect of symbionts on their hosts (Box 1) demonstrates the importance of microbiota in insect community dynamics. On an ecological timescale, symbionts influence the way in which their hosts feed, reproduce, compete, and defend themselves against natural enemies (McLean et al., 2016). Over evolutionary time, these influences may facilitate host species' coexistence, cause localized deterministic extinctions, or impact species coevolutionary dynamics (Frago et al., 2012; McLean et al., 2016). To connect insects, microbiota, and the environment into a wider context, and to consider the importance of horizontal transmission in particular, we advocate a macroecological viewpoint with the dispersal‐led concept of metacommunity theory.

Box 1 **Insect–microbe interactions**

Below, we detail key areas in which symbionts can affect host phenotype, and thus the host's ability to interact with its environment and its community (Cagnolo, Salvo, & Valladares, 2011; Ferrari & Vavre, 2011; McLean et al., 2016).

**Herbivory**

The microbiome affects host‐plant use, as acquisition of novel endosymbionts, or gut microbes, can potentially facilitate species interactions with different plants (Hansen & Moran, 2014; Figure 2a) and the acquisition of novel resources (Hammer & Bowers, 2015). New food sources can change population and community dynamics due to rapid expansion of host populations following sudden resource availability (Frago et al., 2012; Hulcr & Dunn, 2011). Symbionts are also capable of mediating interactions with plants. Frago et al. (2017) found that several endosymbionts reduced parasitoid wasp recruitment by attenuating the release of volatiles from a plant under attack by aphids, further indicating the wide‐reaching role played by host‐associated microbes (also see Cusumano et al., 2018; for viral symbionts).

**Protective symbiosis**

Microbiota have been shown to alter host defense against natural enemies (Imler, 2014; Parker, Spragg, Altincicek, & Gerardo, 2013; Rothacher, Ferrer‐Suay, & Vorburger, 2016; Figure 2b). One of the best studied endosymbionts with regard to parasitoids is the bacterium *Hamiltonella defensa*, which has been demonstrated to provide aphids with protection against parasitoids in the laboratory (Oliver, Russell, Moran, & Hunter, 2003) and in the field (Hrček et al., 2016; Rothacher et al., 2016) by providing phage‐encoded toxins that kill developing parasitoids (Oliver, Degnan, Hunter, & Moran, 2009). Other endosymbionts, including *Regiella insecticola*, *Wolbachia, Spiroplasma,* and *Rickettsia,* also provide their hosts with protection against parasitoids (Fytrou, Schofield, Kraaijeveld, & Hubbard, 2006; Hamilton & Perlman, 2013; Vorburger et al., 2010; Xie, Butler, Sanchez, & Mateos, 2014; Xie et al., 2010), fungi (Łukasik, Guo, Asch, Ferrari, & Godfray, 2013; Parker et al., 2013), nematodes (Haselkorn & Jaenike, 2015; Jaenike, Unckless, Cockburn, Boelio, & Perlman, 2010), and RNA viruses (Cattel, Martinez, Jiggins, Mouton, & Gibert, 2016; Hedges, Brownlie, O'Neill, & Johnson, 2008). Additionally, bacteria from the gut microbiome have been shown to regulate insect immunity (Koropatnick et al., 2004; Round & Mazmanian, 2009), with changes in gut microbiome community composition resulting in demonstrable changes to immunity and host resistance to parasitoids (Chaplinska et al., 2016; Ferguson et al., 2018).

**Thermal tolerance**

Symbionts can both increase and decrease thermal tolerance in a variety of hosts (Bensadia, Boudreault, Guay, Michaud, & Cloutier, 2006; Lazzaro, Flores, Lorigan, & Yourth, 2008; Figure 2c). Heat‐shock tolerance in the whitefly *B. tabaci* increases with reduction in *Rickettsia* numbers and the symbiont‐led expression of genes associated with stress response (Brumin, Kontsedalov, & Ghanim, 2011). Conversely, in *A. pisum, Rickettsia* increases heat tolerance by allowing the aphid to retain a higher percentage of bacteriocytes (Montllor, Maxmen, & Purcell, 2002). Disruption of specific regions of the microbiome (e.g., the gut) can have negative consequences for host thermal tolerance because the gut microbiome has positive influence on induction of thermal tolerance proteins within cells (Henry & Colinet, 2018; Liu, Dicksved, Lundh, & Lindberg, 2014). Heat shock can further affect bacterial density in their hosts, which may lead to increased variation in vertical transmission rates (Hurst, Johnson, Schulenburg, & v d & Fuyama, Y., 2000; McLean et al., 2016; Watts, Haselkorn, Moran, & Markow, 2009). In some cases, insects have lost their endosymbionts completely following sufficiently strong heat‐shock events (Thomas & Blanford, 2003). The sensitivity of bacterial symbionts to temperature suggests that the benefits and costs provided to hosts could be substantially altered in scenarios of significant environmental (Ross et al., 2017) and seasonal (Ferguson et al., 2018) change. These responses require further investigation, especially in the context of changing temperatures predicted to cause increased abiotic stress (Corbin et al., 2017).

**Reproductive manipulation**

Some facultative symbionts (*Wolbachia* and *Spiroplasma*) are known for impacting host reproduction through male‐killing, genetic feminization, and inducing cytoplasmic incompatibility (Harcombe & Hoffmann, 2004; Haselkorn & Jaenike, 2015; Mateos et al., 2006; Montenegro, Solferini, Klaczko, & Hurst, 2005; Werren, Baldo, & Clark, 2008; Xie et al., 2014; Figure 2d). This leads to altered sex ratios in the host population, reducing mating opportunities, and overall population growth rates. *Wolbachia* infection in some insect species has been documented at >90% prevalence, with extreme evolutionary and behavioral consequences (Jiggins, Hurst, & Majerus, 2000). For instance, one study commonly observed *Wolbachia* infections in parasitoid wasps (Vavre, Fleury, Lepetit, Fouillet, & Boulétreau, 1999), and in one species (*Leptopilina heterotoma*), fecundity, adult survival, and locomotor performance were all affected by *Wolbachia* (Fleury, Vavre, Ris, Fouillet, & Boulétreau, 2000). The mechanisms behind *Wolbachia* are still poorly understood (see Jiggins, 2016).

## 4 INTEGRATING METACOMMUNITY THEORY AND INSECT–SYMBIONT STUDIES

Considering interactions and diversity at multiple scales through the prism of metacommunity theory raises new possibilities for the study of insects and their associated microbes. In these networks, each individual host insect harbors its own community of symbionts and gut bacteria. The interactions between bacteria within a host (intrahost) are joined to other hosts (interhost) at larger spatial scales by transmission (i.e., dispersal) of these symbionts, linking individual insects into a metacommunity (Figure 1, Table 1, Box 2; Mihaljevic, 2012). Metacommunity theory will also enable us to account for patch creation, movement, and destruction, as new host insects are born, move, and die (e.g., Box 2). As we discussed above, microbes play vital roles in host biology and mediate interactions throughout the whole community. These same microbes thus alter metacommunity‐level processes through their own vertical and horizontal transmission. The impacts of microbes on their hosts, and their own transmission, can then be modeled as feedback loops to account for biotic changes (Miller et al., 2018). Organizing these systems into a metacommunity framework provides opportunities for us to explore host interactions at a community scale while simultaneously considering the associated symbionts. This will have subsequent benefits for our broader understanding of how symbionts influence host health (Imler, 2014; Parker et al., 2013; Rothacher et al., 2016), how symbionts become contextually detrimental to their hosts, and the circumstances under which hosts eject their symbionts completely (Polin et al., 2014; Vorburger et al., 2013).

One of the most productive ways to implement the metacommunity framework for studying insect–symbiont systems is to use a dual approach that is both mechanism‐based and model‐based, to best explain observable patterns of community assembly, diversity, and abundance. From a modeling perspective, one method for incorporating hosts and symbionts into metacommunities is by adapting models developed to explain the spread of infectious diseases. Seabloom et al. (2015) introduced a flexible mathematical framework to describe pathogen metacommunity dynamics. The model tracks the spread of two infectious agents among host individuals in a population, where hosts can be infected with one or both pathogens, following the standard susceptible‐infectious‐removed (SIR) framework (Anderson & May, 1979; Keeling & Rohani, 2008). While this framework has broad applicability to the study of symbiont metacommunity dynamics, there have been no attempts to guide researchers with regard to integrating these types of models with empirical data. For instance, how do we estimate the key parameters of these models, and how do we test whether our models accurately represent symbiotic systems? In Box 2, we show simple SIR‐type models to explain the vertical and horizontal transmission of symbionts among hosts and assess which processes are most important for explaining patterns of symbiont community composition over space and time. In Box 3, we highlight how conducting experiments with insect model systems will allow us to parameterize these models, and we offer suggestions for how to use data‐model integration to explicitly test metacommunity theory.

One of the issues with studying natural communities (and applying metacommunity theory to natural habitats) is that they rarely have defined boundaries (Leibold et al., 2004). The confinement of microbiota within an insect host is thus advantageous for defining community boundaries in a spatially explicit manner, as the microbiota of an individual represents a single local community (Gucht et al., 2007) and the whole host insect population represents the regional part of the metacommunity (Figure 1 and Table 1, Scenario A). This is significant because the specific definition of “region” strongly influences how patch processes affect metacommunities (Leibold & Chase, 2017; Logue et al., 2011; Moritz et al., 2013). The reduced ambiguity over defined scale (because the local community is the host's microbiota) makes it more straightforward to apply spatially explicit models to these systems. Even with this framework, we can still include the surrounding environment as the metacommunity matrix, thus enabling us to include environment‐sourced horizontal transfer events. One caveat is that, in this proposed insect–microbiome metacommunity, the “patch” (host) is not static in space, so dispersal rates of microbes partly depend on the dispersal of the host. However, spatial frameworks similar to metacommunities (e.g., metapopulation and epidemiological models; Keeling, Bjørnstad, & Grenfell, 2004, and island biogeography; Reperant, 2009) have been successfully applied to systems with mobile hosts. Similarly, the metacommunity framework has been applied to systems without clearly definable patches (Marrec, Pontbriand‐Paré, Legault, & James, 2018). Therefore, it is still possible to match spatial assumptions under these circumstances. Box 2 shows how we can add implicit spatial dynamics into an SIR‐type modeling framework, and how we can start to parameterize these models as well. Other modeling approaches, including probabilistic, event‐driven approaches (e.g., Gillespie's Direct Algorithm, Gillespie, 2007), could also be simulated, and custom model‐fitting code could be generated to fit these stochastic models to experimental or observational time‐series data. This approach could be particularly appropriate for more complex models, where model parameters may have hidden correlations (Kennedy, Dukic, & Dwyer, 2015).

One of the benefits of using metacommunity ecology to study insect–symbiont systems is the flexible use of definitions. As we outline in Table 1, there are multiple scenarios where metacommunity theory can be applied to these systems. The local community scale, especially, can be designated at the discretion of the investigator. We outlined above, and in Figure 1 and scenario A of Table 1, the possibility of treating each individual insect as a local community of bacteria. Below (and in other scenarios of Table 1), we suggest future applications of metacommunity ecology to insect–symbiont systems, including scenarios where symbionts are being actively manipulated as a form of vector control.

Box 2 **A metacommunity model of vertically transmitted symbionts**

Here, we build upon epidemiological models (Anderson & May, 1979; Keeling & Rohani, 2008; Seabloom et al., 2015) to explain the horizontal and vertical transmission of symbionts among insect hosts, and the movement of hosts among habitat patches. Thus, the models capture the dynamics of a simple insect metacommunity, where the dynamics of the symbionts are summarized at the level of a host population, *i*, and host dispersal links all *J* populations in the host metapopulation. We begin with a generalized model framework of two symbionts and one host species:

$$S_{i}^{\prime }=\overset{\text{Within}\;-\;\text{patch dynamics}}{\overbrace{D_{s}\left(V,\theta_{D}\right)+T_{s}\left(V,\theta_{T}\right)}}+\overset{\text{Among}\;-\;\text{patch dynamics}}{\overbrace{M_{s_{i}}\left(S,\theta_{M}\right)}}$$

$$I_{A_{i}}^{\prime }=D_{I_{A}}\left(V,\theta_{D}\right)+T_{I_{A}}\left(V,\theta_{T}\right)+M_{I_{A_{i}}}\left(I_{A},\theta_{M}\right)$$

$$I_{B_{i}}^{\prime }=D_{I_{B}}\left(V,\theta_{D}\right)+T_{I_{B}}\left(V,\theta_{T}\right)+M_{I_{B_{i}}}\left(I_{B},\theta_{M}\right)$$

$$X_{i}^{\prime }=D_{X}\left(V,\theta_{D}\right)+T_{X}\left(V,\theta_{T}\right)+M_{X_{i}}\left(X,\theta_{M}\right)$$

In this set of differential equations, hosts are susceptible (*S*), infected with a single symbiont (*I_A_* or *I_B_*), or coinfected with both symbionts (*X*). The *D*, *T*, and *M* functions represent the dynamics of host demography and vertical symbiont transmission (*D*), horizontal symbiont transmission (*T*), and host migration (*M*). These are functions of the model variables, captured by the vector $V=\left(S_{i},I_{A_{i}},I_{B_{i}},X\right)$, as well as vectors of the respective parameters, stored in ***θ***. Migration is a function of all other subpopulations in the host metapopulation, such that, for example, vector $S=\left(S_{1},S_{2},\ldots ,S_{J}\right)$. This set of differential equations therefore allows for flexibility in defining the specifications of each of the *D*, *T*, and *M* functions. We will use the following expansion of the above equations to suggest a more concrete model of the system.

$$S_{i}^{\prime }=\overset{\begin{array}{c} \text{Host Demography}\\ \text{and Vertical Symbiont Transmission} \end{array}}{\overbrace{\nu_{b}\left[\phi \left(X_{i}+I_{A_{i}}+I_{B_{i}}\right)+S_{i}\right]-\nu_{d}S_{i}}}-\overset{\text{Horizontal Symbiont Transmission}}{\overbrace{\frac{\beta_{A}\left(I_{A}+qX\right)S}{N_{i\left(t\right)}}-\frac{\beta_{B}\left(I_{B}+qX\right)S}{N_{i\left(t\right)}}}}-\overset{\text{Host migration}}{\overbrace{mS_{i}+\Sigma_{l\neq i}^{J}\rho_{i,l}mS_{l}}}$$

$$I_{A_{i}}^{\prime }=\nu_{b}\left[\left(1-\phi \right)\left(c_{A}X_{i}+I_{A_{i}}\right)\right]-\nu_{d}I_{A_{i}}+\frac{\beta_{A}\left(I_{A}+qX\right)S}{N_{i\left(t\right)}}-\frac{\psi \beta_{B}\left(I_{B}+qX\right)I_{A}}{N_{i\left(t\right)}}-mI_{A_{i}}+\sum_{l\neq i}^{J}\rho_{i,l}mI_{A_{l}}$$

$$I_{B_{i}}^{\prime }=\nu_{b}\left[\left(1-\phi \right)\left(c_{B}X_{i}+I_{B_{i}}\right)\right]-\nu_{d}I_{B_{i}}+\frac{\beta_{B}\left(I_{B}+qX\right)S}{N_{i\left(t\right)}}-\frac{\psi \beta_{A}\left(I_{A}+qX\right)I_{B}}{N_{i\left(t\right)}}-mI_{B_{i}}+\sum_{l\neq i}^{J}\rho_{i,l}mI_{B_{l}}$$

$$X_{i}^{\prime }=\nu_{b}\left[\left(1-\phi \right)\left(1-(c_{A}+c_{B}\right))X_{i}\right]-\nu_{d}X_{i}+\frac{\psi \beta_{B}\left(I_{B}+qX\right)I_{A}}{N_{i\left(t\right)}}+\frac{\psi \beta_{A}\left(I_{A}+qX\right)I_{B}}{N_{i\left(t\right)}}-mX_{i}+\sum_{l\neq i}^{J}\rho_{i,l}mX_{l}$$

The model tracks host demography via reproduction and death rates, $\nu_{b}$ and $\nu_{d}$, and we assume that infection with the symbionts does not affect these rates. The model also incorporates vertical transmission of the symbionts. The parameter ϕ is the fraction of births that result in fully symbiont‐free, susceptible hosts, while 1-ϕ is the likelihood of vertical transmission occurring. Parameters $c_{A}$ and $c_{B}$ are the conditional likelihoods of coinfected hosts reproducing and leading to singly infected offspring, assuming they produce offspring with any infection. The term $1-\left(c_{A}+c_{B}\right)$ is therefore the probability of producing coinfected offspring, again conditional on producing offspring with any infection, $\left(1-\phi \right)$.

We assume horizontal transmission occurs in a frequency‐dependent manner via contact between susceptible and infectious hosts (sensu Seabloom et al., 2015), such that the transmission rates for each symbiont, $\beta_{A}$ and $\beta_{B}$, are divided by the habitat patch‐ and time‐specific population size $N_{i\left(t\right)}$. Population sizes within a host habitat patch may fluctuate over time due to within‐patch demography and among‐patch migration. The likelihood of singly infected hosts becoming coinfected is mediated by the infected hosts' susceptibility to a secondary infection, ψ. Susceptible hosts can be infected by single‐ or coinfected hosts, and the transmissibility of symbionts from coinfected hosts is modulated by q, but we assume coinfection occurs sequentially (i.e., a host first becomes infected with one symbiont, then the other).

Host migration occurs when hosts emigrate from the patch, at a per‐capita rate m, or when hosts immigrate to patch i from other patches. The probability of migration from patch l to patch i, $\rho_{i,l}$, can then be a function of the distance between patches $d_{i,l}$. And, importantly, the sum $\Sigma_{l\neq i}^{J}\rho_{l,i}=1$ so that all individuals emigrating from a patch eventually end up in some other patch.

**Addressing metacommunity questions with the model**

Although this model seems complex, it could be quite useful for both theoretical explorations and empirical tests of metacommunity theory (e.g., Box 3). For instance, analytic and numerical model analysis could reveal how the likelihoods of vertical and horizontal transmission affect local and regional coexistence of symbionts in the context of host migration between habitat patches. Additionally, the roles of trade‐offs in symbiont coexistence could be analyzed, such as trade‐offs in the host traits (e.g., demography and migration) compared to trade‐offs in the symbiont traits (e.g., rates of vertical and horizontal transmission). Furthermore, in Box 3 we demonstrate how this model could be parameterized with empirical studies of insect–symbiont systems. The parameterized models can then be used to determine how well model predictions match observed patterns of symbiont community composition across space. Thus, insect–symbiont systems could be used to rigorously test the role of metacommunity dynamics in structuring symbiont communities.

Box 3 **Integrating theory and empirical data to understand metacommunity dynamics**

There have been few attempts to guide researchers with advice for integrating metacommunity models with empirical data. This process is critically important to test whether metacommunity theory can explain patterns of symbiont community composition across space and time, and more specifically to explore which local and regional processes are most important for explaining these patterns. Parameterized models can also be used to make forecasts which can be useful, for instance, in the microbial control of insect populations. Here, we briefly highlight methods of model parameter estimation using laboratory experiments and offer suggestions for how to use data‐model integration to test metacommunity theory with insect model organisms. Our goal is to emphasize the utility of insect–symbiont systems for understanding the applicability of metacommunity theory to communities of host‐associated microorganisms. Supplemental code for model fitting is provided. We note that our methods rely on longitudinal sampling of host populations, but other methods of estimating transmission do not rely on taking multiple samples through time (Dwyer, Elkinton, & Buonaccorsi, 1997), but are perhaps less generalizable.

**Introduction to model fitting for parameter estimation**

**Figure 3:**
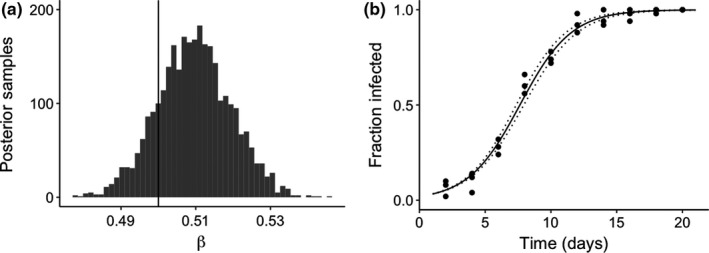
Graphs represent fitting a simple susceptible‐infected (SI) model to hypothetical experimental data. In this experiment, a single‐infected host was released in a population of 49 susceptible hosts, and this was replicated across three host populations. Symbiont transmission occurs horizontally, from infected individuals to susceptible individuals. We simulated the data based on the SI model, adding observation error, and setting the transmission rate to 0.50 day^−1^ host^−1^. The model was then fit to the synthetic data with *Stan* using 3 Hamiltonian Monte Carlo chains, with a 2,000 iteration warm‐up period, and 5,000 total iterations, thinning by 3. A vague prior (*N*(0, 5)) was used for the transmission rate. (a) Marginal posterior estimate of transmission rate, with vertical line delineating the true parameter value (0.50). (b) Fit of the model (median and 95% credible interval) to time‐series data of the fraction of the population infected, where the three populations were sampled every 2 days of the experiment

To begin parameterizing the equations in Box 2, we deal with horizontal transmission, which is arguably the most complex dynamic. We must first measure the transmission rates of each symbiont. One approach to estimate transmission rate is to conduct a simple laboratory experiment in which the researcher releases infected hosts into a population of susceptible (uninfected) hosts and documents the change in prevalence over time (Table 1, Figure 3). Then, the researcher can fit a simplified SIR model to these data to estimate transmission rates. We assume the dynamics of the experimental system can be represented by the simple equation:

$$I^{\prime }=\beta I\left(N-I\right)/N.$$

In this differential equation model, we assume that a host population of constant size N is made of susceptible hosts (S) and infected hosts (I), such that N=S+I. The rate of change in the infected class is mediated by the transmission rate β and contact between susceptible (N-I) and infectious hosts. If we experimentally expose a known number of susceptible hosts to a known number of infectious hosts, we can track the proportion of hosts that become infected over time. We can then fit this simple dynamical system to the experimental data. Specifically, we compare the fraction of the experimental host population infected at any given time point to the fraction infected in our model, and we can assume the likelihood of the data $P\left(D|\beta \right)$ follows a binomial probability distribution (Figure 3). This can be done in a Bayesian framework, for instance, by fitting the differential equation model to the data in *Stan*, an open‐source statistical programming language (Carpenter et al., 2017). This same model‐fitting routine can be used for more complex SIR‐type models (e.g., below).

**Multisymbiont model and experiments**

To continue parameterizing the equations in Box 2, we must understand how multiple symbionts interact in the system. We can simplify the model to only include horizontal transmission to encompass the dynamics of an experiment that occurs on a timescale with no host demography, and in which migration is not allowed.

$$S^{\prime }=-\frac{\beta_{A}\left(I_{A}+qX\right)S}{N}-\frac{\beta_{B}\left(I_{B}+qX\right)S}{N}$$

$$I_{A}^{{}^{\prime }}=\frac{\beta_{A}\left(I_{A}+qX\right)S}{N}-\frac{\psi \beta_{B}\left(I_{B}+qX\right)I_{A}}{N}$$

$$I_{B}^{{}^{\prime }}=\frac{\beta_{B}\left(I_{B}+qX\right)S}{N}-\frac{\psi \beta_{A}\left(I_{A}+qX\right)I_{B}}{N}$$

$$X^{\prime }=\frac{\psi \beta_{B}\left(I_{B}+qX\right)I_{A}}{N}+\frac{\psi \beta_{A}\left(I_{A}+qX\right)I_{B}}{N}$$

Although this model seems complex, there are only four parameters, two of which (the transmission rate of symbiont A, $\beta_{A}$, and the transmission rate of symbiont B, $\beta_{B}$) can be estimated with the experiment outlined above. Therefore, we can conduct another experiment to estimate the remaining two parameters. And when we use Bayesian inference, we can use prior probability distributions for $\beta_{A}$ and $\beta_{B}$ derived from the single‐symbiont experiments.

**Figure 4:**
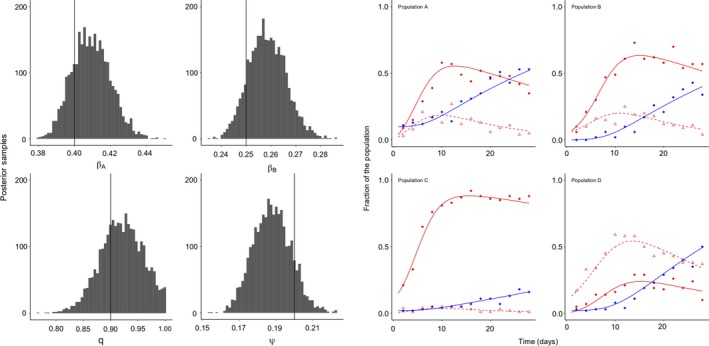
Fitting the two symbionts—one host species SI model to synthetic data, from Box 2 “Multisymbiont model and experiment” section. Four populations of 100 hosts were exposed to variable initial numbers of hosts infected with symbiont A (closed red circles, red line), symbiont B (open red triangles, dashed red line), or coinfected with both symbionts (closed blue circles, blue line). Experimentally manipulating the initial conditions enables us to estimate the parameters with more power, because we observe more variable dynamics in the system. Specifically, the initial conditions for each simulated population ($S\left(0\right),I_{A}\left(0\right),I_{B}\left(0\right),X\left(0\right)$) are as follows: (a) 90, 0, 0, 10; (b) 90, 5, 5, 0; (c) 88, 10, 0, 2; (d) 88, 0, 10, 2. We chose these values to demonstrate that the transient dynamics of the model are influenced by subtle changes to initial conditions, and we should see these dynamics reflected in the experimental data. Again, the model was fit to the synthetic data with *Stan* using vague priors for each of the four parameters, and 5,000 total sampling iterations. Graphs in the left‐hand panel show the marginal posterior samples for each parameter, with the vertical line delineating the true parameter value. To reiterate, the parameters are as follows: $\beta_{A}$ and $\beta_{B}$ are the transmission rates of the two symbionts, respectively; q modulates the likelihood that susceptible hosts become infected through contact with coinfected hosts (i.e., q=1 would mean that there was an equal likelihood of susceptible hosts being infected by single‐ or coinfected hosts); and ψ modulates the likelihood that single‐infected hosts will become coinfected by a secondary symbiont. Graphs in the right‐hand panel depict the simulated, synthetic data, where the fraction of hosts infected with one or both pathogens changes over time. The lines represent the median model predictions. Only median posterior model predictions are shown, for clarity

In a multisymbiont experiment, we can create experimental populations of hosts, and we can expose these populations to varying numbers of single‐ or coinfected hosts. We again track how the fractions of single‐ and coinfected hosts change over time, as the symbionts spread. We construct a likelihood function that compares the model's predicted number (or fraction) of hosts in each class to the experimentally derived numbers. By altering the starting conditions (i.e., the initial numbers of susceptible, singly and coinfected hosts), we gain more power to estimate the parameters, allowing for estimation of all four parameters from a small number of experimental populations (Figure 4).

**Host demography, vertical transmission, and spatial processes**

We do not spend much time on measuring the parameters of host demography or vertical transmission in the equations in Box 2. First, empirically estimating the rates of host demography in ecological models has been covered in great detail (McCallum, 2008). In addition, the parameters of vertical transmission could be easily measured by determining the probability of singly and coinfected hosts producing singly or coinfected offspring, or fully susceptible offspring. Measuring the rates of host migration can admittedly be complex, but will likely be simpler for insect model organisms (Table 1). Mark–recapture studies, for example, have been used to estimate mosquito dispersal rates for decades (e.g., Reisen et al., 1991). Therefore, emigration rates and quantitative dispersal kernels could be parameterized by determining the probabilities of short‐range and long‐range movements in the laboratory and/or in the field.

**Model comparisons to test metacommunity theory**

The examples above assume that the mathematical model presented in Box 2 is an appropriate representation of the system's dynamics. However, this is not necessarily true. In other words, the applicability of metacommunity theory to a particular system is a testable hypothesis. We can construct different versions of our mathematical models, including or excluding specific assumptions and processes, and then fit these models to our time‐series data. We can then use formal model‐comparison approaches (Hooten & Hobbs, 2014; Vehtari, Gelman, & Gabry, 2016) to determine which models best explain observational data. For instance, we can collect data from the field on how the composition of the symbiont community changes through time in a host metapopulation. By comparing how different metacommunity models fit to these data, we can therefore test which local and regional mechanisms are most important.

In summary, integrating time‐series data and model‐fitting approaches can expand our understanding of metacommunity dynamics. Furthermore, insect–symbiont communities are unique and experimentally tractable model systems for exploring the applicability of metacommunity theory to host‐associated microbial communities (Table 1).

## 5 DIRECT APPLICATION OF INSECT–MICROBIOTA METACOMMUNITIES

A direct way to study dispersal in an insect–microbiome metacommunity could be to focus on horizontal transmission of facultative symbionts throughout a host–parasitoid community, as horizontal acquisition of symbionts can be key for host survival against natural enemies (Haselkorn et al., 2009; Jaenike, 2009; Moran & Dunbar, 2006). One way to investigate this experimentally would be to use hosts that are axenic (devoid of all bacteria) or gnotobiotic (possessing select microbiota only) before initiating colonization with a community of bacteria, then allowing dispersal across the host community to occur (Table 1, Scenario A) by introducing parasitoids to facilitate the spread of bacteria, for instance (the “dirty needle effect”; see section on “Horizontal transmission”). This could be expanded upon by measuring symbiont dispersal in conjunction with other effects. For example, symbiont dispersal under different temperature regimes will provide information on how host–symbiont metacommunities might respond to a changing climate, and thus, how they would be expected to affect host performance (Corbin et al., 2017; Feldhaar, 2011). A similar experimental approach for insect–microbiota metacommunities is to determine the effects of disturbance on stability and interactions within the metacommunity by feeding hosts with antibiotics. These synthetic metacommunities will also reveal the effects that changes in microbiome (local community) diversity have on the local community structure (Adair & Douglas, 2017) and regional host community structure, with subsequent possibilities for relating structure to metacommunity stability through these local manipulations (Leibold et al., 2004; Loreau, 2010).

**Table 1:** Suggested scenarios for the application of metacommunity theory to insect–symbiont systems, taking into consideration community definitions, the possible questions that could be addressed with each system, and outlining a potential experiment to test address the question

Scenario	Local community	Regional community	Question(s) addressed	Experimental outline	Metacommunity response variable
A (see also Figure 1)	Individual insect	Host insect community	How much horizontal transmission of bacteria between individual insects occurs over a single host generation? How do abiotic factors or variable parasitoid pressure influence horizontal transmission?	Introduce a target bacterium to a metacommunity of axenic insects, and sample them at the end of one host generation to see how much the target bacterium has spread via horizontal transmission	Individual insect microbiome (local community) diversity
B	One insect host species	Multiple insect host species	What barriers exist between species preventing horizontal transmission of symbionts? (e.g., Is coevolution of host and symbiont a predominant barrier preventing horizontal transmission from one host species to another?)	Experimentally, again with axenic hosts, one could introduce a symbiont in different ‘doses’ to determine the point where dispersal is sufficient to overcome natural dynamics	Microbiome (local community) diversity
C	One individual plant	Multiple plants of single or multiple species, with their insect pests and symbionts included	How does a spatially structured metacommunity change the dynamics of herbivore–symbiont dispersal? Metacommunity structured by the location of plants, with parameters changed relative to previous scenarios by plants not moving and having much longer life spans	Comparison of different plant spatial configurations with measures of herbivore density, the number of symbionts, and the dispersal of symbionts, as a result of the distance between plant‐associated communities	Diversity of insects and associated symbionts on a particular plant
D	All insects associated with one plant individual	All insects associated with multiple plant individuals	How much does pest dispersal facilitate symbiont movement between plants? This scenario is a combination of **scenarios B and C**, based on the coevolved barriers between insect species and their impacts on symbiont dispersal, and the plant‐focused spatially structured metacommunity	Dispersal measured as the movement of insect herbivores (e.g., aphids) between plants, and the subsequent impacts on symbiont dispersal within the metacommunity (see Brady et al., 2014; Frago et al., 2017 for the associations between symbiont, insect, and plant)	Diversity of insects and associated symbionts on one particular plant
E	One local site of a focal symbiont‐infected host species, and close relative species of the host	Multiple sites of the focal insect host, its symbiont, and closely related species	Which insect species does a biocontrol symbiont spread to within a wild community? Will other species in the microbiome of wild hosts facilitate establishment of *Wolbachia*? This is a specific application toward biocontrol efforts. The example presented is the attempt to use male‐killing strains of *Wolbachia* to reduce populations of the dengue fever mosquito (*Aedes aegypti*)	In this scenario, dispersal is a combination of the mosquito's movement, transmission of the symbiont, and establishment of the symbiont, measured over time and space by capturing individuals of *A. aegypti* (and closely related species) and measuring them for the used *Wolbachia* strain. This enables us to quantify dispersal distance over time, and simultaneously consider spillover events into other insects in the natural community	Insect microbiome diversity

Theoretical metacommunity models, like those shown in Boxes 2 and 3, have the potential to identify the most important factors in insect–microbiome metacommunity assembly by fitting alternative models to experimental data. Modeling metacommunities can also deepen our understanding patterns of diversity of host‐associated microbiomes. Previous work on microbiomes has suggested that stochasticity plays a significant role in community assembly, and that the process is inherently hard to predict (see Adair, Wilson, Bost, & Douglas, 2018; Obadia et al., 2017; Sieber et al., 2019; Vega & Gore, 2017), based on findings that are consistent with the neutral theory of biodiversity (Hubbell, 2001). Recent models for metacommunity diversity (e.g., O'Sullivan, Knell, & Rossberg, 2019) can be utilized to answer questions about ecological structural stability influencing microbiome diversity, and whether the microbiome adheres to broad ecological patterns of diversity. For instance, testing whether symbiont communities fit the species‐abundance distribution (SAD) or species‐area relation (SAR). The aforementioned studies indicating that stochasticity plays a prominent role in microbiome composition would appear to infer that diversity patterns in microbiomes differ from those observed elsewhere in ecology. Thus, a pressing question in microbial ecology is to determine whether patterns of microbial community composition are driven by the same mechanisms that drive patterns of free‐living community composition. More work is required to unravel microbiome diversity, and metacommunity modeling is a potential avenue to further explore this aspect of microbiomes.

Another potential application for metacommunity theory and insect–symbiont systems is to improve understanding of symbiont dynamics in scenarios where symbionts are being utilized for human benefit (Table 1, Scenario E). A prominent example is the use of *Wolbachia* to manipulate host sex ratios as a form of biocontrol against undesirable species (Hoffmann et al., 2015), particularly disease‐spreading mosquitoes such as *Aedes aegypti* (Frentiu et al., 2014; Ross et al., 2017). One of the most important aspects for releasing *Wolbachia‐*infected mosquitoes is knowing how they will disperse, both in terms of how the infected hosts will move and how the wild symbiotic communities will respond to *Wolbachia* introduction. The structure of their dispersal routes is crucial for infected mosquitoes to access wild insect communities and for *Wolbachia* to disperse. An equally important aspect of *Wolbachia* dispersal is understanding how *Wolbachia* will interact with other endosymbionts and the gut microbiome (see subsection “Interactions within microbial communities”). One possibility could be to aid *Wolbachia* dispersal via facilitation from another symbiont. In addition, we also need to understand symbiont dynamics for scenarios where a host becomes a pest species due to protective symbiosis (McLean et al., 2016). To counteract pests with biocontrol, we need to know the best potential control option, and therefore must know which enemies can be countered with protective symbionts and how these symbionts disperse throughout the host population (e.g., if applying a parasitoid for biocontrol of a pest risks facilitating defensive symbiont dispersal via the dirty needle effect). Using the metacommunity framework to explicitly measure symbiont dispersal within a community‐wide context could provide new insights into currently unexplained patterns, such as the lack of phylogenetic clustering exhibited by *Wolbachia* and other symbionts in their host species (Henry et al., 2015; Smith et al., 2012).

## 6 CONCLUSION

Strong evidence that host‐associated microbiota influence interactions among their hosts warrants greater consideration of the mechanisms that drive symbiont diversity in large‐scale studies, and we propose metacommunity theory as a framework to achieve this. We recommend that insect–microbiota model systems be used to investigate the role of symbionts in shaping host interactions within metacommunities, the importance of phenotype transfer as a result of symbiont dispersal, and the ecological consequences of symbiont transmission. Through the microbial prism, we are likely to achieve greater understanding of the mechanisms that influence metacommunities and the dynamic processes within them.

## CONFLICT OF INTEREST

The authors declare no known conflict of interest regarding the publication of this manuscript.

## AUTHOR CONTRIBUTIONS

JJB, JRM, LDM, and JH contributed to development of the ideas and the writing of the manuscript.

## Data Availability

Model code is included as Supporting Information.
